# The role of endoscopy and findings in COVID-19 patients, an early North American Cohort

**DOI:** 10.1186/s12876-021-01796-4

**Published:** 2021-05-07

**Authors:** Gabriela Kuftinec, B. Joseph Elmunzer, Sunil Amin, North American Alliance for the Study of Digestive Manifestations of Covid-19, Joseph Elmunzer, Rebecca L. Spitzer, Lydia D. Foster, Ambreen A. Merchant, Eric F. Howard, Vaishali A. Patel, Mary K. West, Emad Qayad, Rosemary Nustas, Ali Zakaria, Marc S. Piper, Jason R. Taylor, Lujain Jaza, Nauzer Forbes, Millie Chau, Luis F. Lara, Georgios I. Papachristou, Michael L. Volk, Liam G. Hilson, Selena Zhou, Vladimir M. Kushnir, Alexandria M. Lenyo, Caroline G. McLeod, Sunil Amin, Gabriela N. Kuftinec, Dhiraj Yadav, Charlie Fox, Jennifer M. Kolb, Swati Pawa, Rishi Pawa, Andrew Canakis, Christopher Huang, Laith H. Jamil, Andrew M. Aneese, Benita K. Glamour, Zachary L. Smith, Katherine A. Hanley, Jordan Wood, Harsh K. Patel, Janak N. Shah, Emil Agarunov, Amrita Sethi, Evan L. Fogel, Gail McNulty, Abdul Haseeb, Judy A. Trieu, Rebekah E. Dixon, Jeong Yun Yang, Robin B. Mendelsohn, Delia Calo, Olga C. Aroniadis, Joseph F. LaComb, James M. Scheiman, Bryan G. Sauer, Duyen T. Dang, Cyrus R. Piraka, Eric D. Shah, Heiko Pohl, William M. Tierney, Stephanie Mitchell, Ashwinee Condon, Adrienne Lenhart, Kulwinder S. Dua, Vikram S. Kanagala, Ayesha Kamal, Vikesh K. Singh, Maria Ines Pinto-Sanchez, Joy M. Hutchinson, Richard S. Kwon, Sheryl J. Korsnes, Harminder Singh, Zahra Solati, Amar R. Deshpande, Don C. Rockey, Teldon B. Alford, Valerie Durkalski, Field F. Willingham, Patrick S. Yachimski, Darwin L. Conwell, Evan Mosier, Mohamed Azab, Anish Patel, James Buxbaum, Sachin Wani, Amitabh Chak, Amy E. Hosmer, Rajesh N. Keswani, Christopher J. DiMaio, Michael S. Bronze, Raman Muthusamy, Marcia I. Canto, V. Mihajlo Gjeorgjievski, Zaid Imam, Fadi Odish, Ahmed I. Edhi, Molly Orosey, Abhinav Tiwari, Soumil Patwardhan, Nicholas G. Brown, Anish A. Patel, Collins O. Ordiah, Ian P. Sloan, Lilian Cruz, Casey L. Koza, Uchechi Okafor, Thomas Hollander, Nancy Furey, Olga Reykhart, Natalia H. Zbib, John A. Damianos, James Esteban, Nick Hajidiacos, Melissa Saul, Melanie Mays, Gulsum Anderson, Kelley Wood, Laura Mathews, Galina Diakova, Molly Caisse, Lauren Wakefield, Haley Nitchie

**Affiliations:** 1 Division of Digestive and Liver Diseases, University of Miami Miller School of Medicine, Miami, FL USA; 2 Division of Gastroenterology and Hepatology, Medical University of South Carolina, Charleston, SC USA; 3 Department of Medicine, Division of Gastroenterology, University of Miami, Leonard M. Miller School of Medicine, 1120 NW 14th Street, Clinical Research Building, Suite 1116, Miami, Fl 33136 USA

**Keywords:** SARS-CoV-2, Endoscopy, Viral Injury, GI bleeding, Enteral access

## Abstract

**Background and aims:**

Gastrointestinal manifestations in patients with COVID-19 are common but the role of endoscopy in this patient population remains unclear. We investigated the need for endoscopic procedures, their findings, and impact on patient care in a systematic and geographically diverse sample of patients hospitalized with COVID-19.

**Methods:**

As part of the North American Alliance for the Study of Digestive Manifestations of COVID-19, we identified consecutive patients hospitalized with COVID-19 at 36 medical centers in the USA and Canada. We performed a secondary analysis of patients who underwent endoscopy, collecting information on endoscopic indications, findings, interventions, staffing, procedure location, anesthesia utilization, and adverse events.

**Results:**

Data were collected on 1992 patients; 24 (1.2%) underwent 27 endoscopic procedures (18 upper endoscopies, 7 colonoscopies, 2 endoscopic retrograde cholangiopancreatographies). The most common indications were: gastrointestinal bleeding (13) and enteral access (6). The most common findings were erosive or inflammatory changes. Ten patients underwent an endoscopic intervention for hemostatic therapy (2), enteral access (6), or biliary obstruction (2). Half of cases employed anesthesiology support; no sedation-related adverse events were reported. One-third of cases were performed in the intensive care setting and one quarter in the endoscopy unit.

**Conclusions:**

In this large, systematic, geographically diverse cohort of patients hospitalized with COVID-19 in North America, very few patients underwent endoscopy despite a high prevalence of gastrointestinal manifestations. Almost all endoscopic findings and interventions were thought related to critical illness rather than direct viral injury. This systematic assessment of endoscopic necessity and outcomes may help guide resource allocation in the event of ongoing and future surges.

## Introduction

Severe acute respiratory syndrome coronavirus 2 (SARS-CoV-2), the virus responsible for COVID-19, has caused a global pandemic, with over 28 million cases in the United States alone and over 114 million cases worldwide as of March 1, 2021 [1]. Although primarily a respiratory virus, SARS-CoV-2 causes several gastrointestinal (GI) symptoms with a reported prevalence of 53% in hospitalized patients [2]. In fact, the first patient with COVID-19 in the US presented with 2 days of cough, fatigue, nausea and vomiting followed by diarrhea, and was diagnosed via a stool specimen [3]. The presence of GI symptoms was initially associated with worse clinical outcomes, including higher mortality [4], however, recent data have contradicted initial observations [2]. While guidelines are available for the management of pneumonia in patients with COVID-19, there is little consensus on how to best approach GI disorders [5].

A principal uncertainty is the role of endoscopy in patients with COVID-19. A case report early in the pandemic highlighted that gastrointestinal symptoms may be the presenting expression of COVID-19 and suggested the possibility of ischemic colitis as a direct consequence of SARS-CoV-2 infection [6]. A series from China around this same time demonstrated SARS-CoV-2 viral RNA in biopsy specimens obtained from the foregut and rectum of infected patients [7]. Subsequent case series, however, have indicated that endoscopic findings in COVID-19 patients are likely to reflect critical illness rather than the direct effect of a GI-tropic virus. In a series from Lombardy, Italy of 38 patients who underwent endoscopic evaluation, 37% had esophagitis, peptic ulcer, or erosive gastritis, but notably 5 patients (13%) had ischemic or hemorrhagic colitis [8]. A multicenter series from New York City comprising 84 COVID-19-positive cases similarly identified esophagitis, peptic ulcer disease, or gastritis in 31% of cases and colitis in 8% [9]. A recent analysis of COVID-19 patients with gastrointestinal bleeding also largely observed lesions that were considered to be related to critical illness, such as gastroduodenal and rectal ulcers, rather than clear viral injury [10].

In all these studies, however, the denominator population from which study samples originated was not reported and therefore the burden of endoscopic utilization in COVID-19 patients remains unclear. Additionally, existing studies reflect the endoscopic experience of a specific geographic region and more generalizable data from varying geographic regions and practice settings would be informative. Lastly, information on anesthesia utilization, anesthesia-related adverse events, procedure environment and staffing in this patient population has not been widely reported and would be of value.

Since endoscopy is dangerous in patients with respiratory compromise and/or critical illness and may pose a threat to health care workers by possible aerosolization of respiratory particles and exposure to gastrointestinal droplets, an expanding understanding of the importance and safety of endoscopy in patients with COVID-19 is necessary to further refine our assessment of its risk–benefit ratio. Therefore, the aims of our study were: (1) to estimate the burden of endoscopic utilization in a systematic cohort of patients; (2) to further describe endoscopic findings and the impact of endoscopy in COVID-19 patients across a large and geographically diverse network of medical centers in North America; and (3) to explore anesthesia utilization and adverse events as well as procedure environment and staffing in infected patients.

## Materials and methods

As part of the North American Alliance for the study of Digestive Manifestations of COVID-19, this was an observational cohort study conducted across 36 medical centers in the Unites States and Canada. Institutional Review Board (IRB) approval was secured at each participating institution and deemed that informed consent for each individual patient not needed given the retrospective, chart review nature of the study design. Each center aimed to enroll the first 50–100 consecutive adult patients hospitalized with confirmed SARS-CoV-2 infection.

We collected demographic, clinical, laboratory, imaging, and endoscopic data from the time of symptom onset until discharge, death, or the end of the study period for each included patient. Data were manually collected by study personnel (clinical research coordinators, medical students, trainees, and/or attending gastroenterologists) under the oversight of a designated clinician-investigator. Accurate and consistent data collection across a large network of centers was ensured in several ways, summarized as follows: (1) the data coordinating center provided formal instructions and a manual of procedures for data abstraction, which were reinforced by frequent communications between the sites and coordinating center; (2) a dedicated data manager at the coordinating center reviewed incoming data for missing or duplicate data, values outside of accepted boundaries, and discrepant or conflicting responses; and (3) data were reviewed in aggregate by the study team to assess for inconsistencies and outliers by center. All methods were performed in accordance with relevant guidelines and regulations at each participating institution.

In this secondary analysis, we specifically focused on patients who underwent an endoscopic procedure. For this subgroup of patients, sites were asked to complete a supplemental data collection form that included information on the type of procedure, indication, findings, assessment of attribution of the findings to critical illness or COVID-19, personnel involved in and location of procedure, as well as type of anesthesia employed, and adverse events related to anesthesia. Descriptive Statistics were used to report our findings.

## Results

### Patients

One thousand nine hundred and ninety-two patients between were included between April 15 and June 5, 2020. 242 patients had a gastroenterology consult called during their inpatient hospitalization (12%). Of the total 1992 patients, 24 (1.2%) underwent a total of 27 endoscopic procedures. The mean age of these patients was 65 years. The average Body Mass Index (BMI) was 29 mg/kg^2^. The majority (83.3%) were men; 9 were black, 10 white, 1 was Asian, and race was unknown in the others. All 24 patients who underwent endoscopy were admitted to the intensive care unit during their hospital course; 20 required mechanical ventilation and 16 required vasopressor support. The majority of patients (18, 75%) received COVID-19 directed treatment, including 16 with Hydroxychloroquine, 4 with Tocilizumab, and 3 with Remdesivir. Seven patients died. Full demographic information is shown in Table 1. All procedures were conducted as inpatients during hospital admission.

**Table 1:** Demographics of COVID positive patients undergoing endoscopic procedures

Demographics		
Age, mean (SD)	65.2 (11.2)	
Sex, n (%)	Male	20 (83.3)
	Female	4 (16.7)
Race, n (%)	White	10 (41.7)
	Black	9 (37.5)
	Asian	1 (4.2)
	Unknown	4 16.7)
Ethnicity, n (%)	Hispanic	3 (12.5)
	Not Hispanic	20 (83.3)
	Unknown	1 (4.2)
Health care worker, n (%)	0 (0)	
COVID-19 treatments		
Remdesivir, n (%)	3 (12.5)	
Hydroxychloroquine, n (%)	16 (66.7)	
Tocilizumab, n (%)	4 (16.7)	
Hospital related outcomes		
Intensive care admission, n (%)	24 (100)	
Mechanical ventilation, n (%)	20 (83.3)	
Vasopressor support, n (%)	16 (66.7)	
Death, n (%)	7 (29.2)	

### Procedure type, indications, and findings

Eighteen of the 24 patients underwent upper endoscopy, 7 underwent colonoscopy, and 2 underwent endoscopic retrograde cholangiopancreatography (ERCP). Three patients underwent multiple endoscopic procedures on the same day (upper endoscopy followed by colonoscopy). Endoscopic indications and findings are listed in Table 2.

**Table 2:** Endoscopic data

Procedure				
Endoscopy, n (%)	18 (75)			
Colonoscopy, n (%)	7 (29.2)			
ERCP, n (%)	2 (8.3)			
Type of sedation, n (%)	Conscious			6 (25)
	MAC			8 (33.3)
	General			10 (41.7)
Reason for endoscopy				
Need for enteral access, n (%)	6 (25)			
Overt Bleeding, n (%)	13 (54.2)			
Anemia without overt bleeding, n (%)	1 (4.2) (OGT placed during EGD as well)			
Biliary decompression, n (%)	2 (8.3)			
Diarrhea, n (%)	1 (4.2)			
Obstruction, n (%)	1 (4.2)			
Need for intervention, n (%)	GI Bleed		2 (8.3)	
	Enteral feeding access		6 (25)	
	Biliary decompression		2 (8.3)	
Endoscopic findings				
Endoscopy n = 18, n (%)	Enteral tube placement		6 (33.3)	
	Inflammation/ulcers		10 (55.6)	
	AVM		1 (5.6)	
	Varices		1 (5.6)	
	Normal		1 (5.6) (one for OGT placement)	
Colonoscopy n = 7, n (%)	Diverticulosis		2 (8.6)	
	Hemorrhoids		3 (42.9)	
	Rectal ulcer		1 (14.3)	
	Blood without source		1 (14.3)	
	Normal		1 (14.3)	
ERCP n = 2, n (%)	Choledocholithiasis		1 (50)	
	Biliary Sludge		1 (50)	
Related to COVID, n (%)	Yes	2 (8.3)		
	No	22 (91.7)		

Of the 18 upper endoscopies, fourteen were performed for suspected blood loss anemia: 13 with and 1 without overt bleeding. Six were performed to place an orogastric tube (OGT) or percutaneous endoscopic gastrostomy tube placement for enteral access. Of the 18 upper endoscopies, ten procedures identified esophagitis [4], gastritis [2], or ulcerations in the upper GI tract [6] that did not require intervention. One identified a bleeding duodenal lesion (described as an arteriovenous malformation) that required clip placement. One patient had bleeding esophageal varices that required band ligation and one upper endoscopy was reported as normal.

Of the 7 colonoscopies, 5 were performed for overt bleeding, one for colonic obstruction, and one for diarrhea. Two procedures identified diverticulosis, 3 identified hemorrhoids, one a rectal ulcer due to fecal management system, and one demonstrated blood throughout the colon without a source identified (suspected small bowel source). One colonoscopy was normal.

Both ERCPs were successfully performed for biliary obstruction; one demonstrating choledocholithiasis and the other bile duct sludge.

In the majority of patients (22, 91.7%), the endoscopist deemed the endoscopic findings to be more likely related to prolonged hospitalization or critical illness rather than COVID-19. Only 1 patient (4.2%) had an endoscopic finding thought to be related to the virus itself: erosive gastritis. A second patient had a normal colonoscopy but on cross-sectional imaging was noted to have pneumatosis intestinalis. No major procedure-related adverse events were reported. There were 2 reported cases (8.3%) mucosal trauma from OGT placement.

### Anesthesia type, procedure location, timing and staffing

Nine patients were mechanically ventilated prior to their endoscopic procedure. These patients and one additional patient received general anesthesia. Eight patients received monitored anesthesia care and 6 received conscious sedation. No patients experienced adverse cardiopulmonary events directly related to sedation for their endoscopic procedure.

**Fig. 1:**
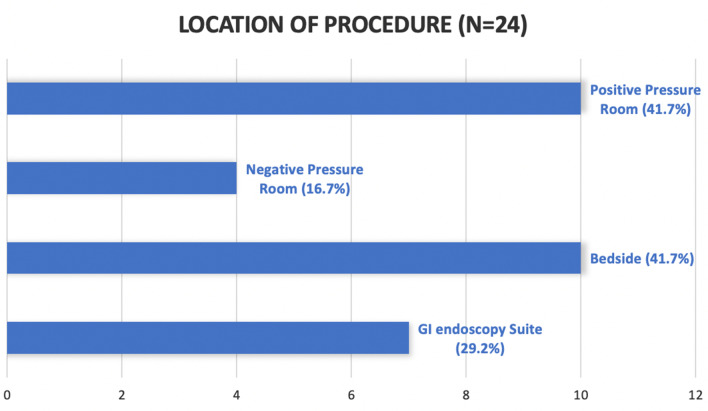
Procedure location used for endoscopic procedure

Ten of the total 24 patients that underwent at least one endoscopic procedure (41.7%) had their procedure performed in the bedside (ICU) setting, which were not specified as positive or negative pressure rooms. 7 (29.2%) patients had their procedure performed in the endoscopy unit. 4 procedures (16.7%) took place in a negative pressure room, 2 in the operating room, one within the endoscopy suite and one in a special COVID procedural room. 10 cases (41.7%) took place in positive pressure rooms, 4 outside the endoscopy suite and 6 within the endoscopy suite (Fig. 1).

Eight patients underwent the endoscopic procedure the same day that the in-hospital consult was placed. Nine patients underwent their endoscopic procedure the day after the initial consult, two within 2–3 days and 5 more than 6 days from the date of the initial consult.

**Fig. 2:**
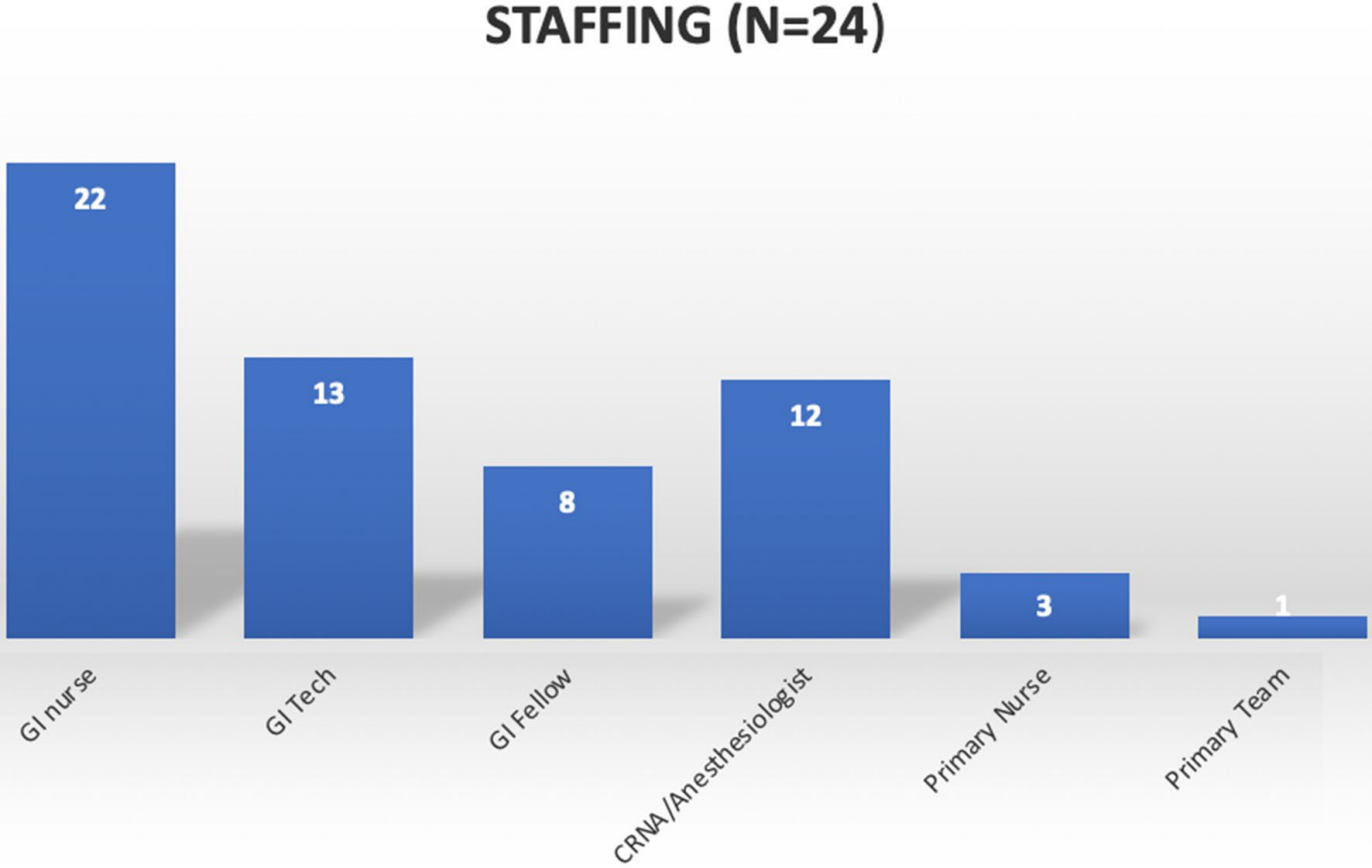
Procedure staffing by staff type

The large majority of patients (22/24; 91.7%) underwent procedures that involved an endoscopy nurse in the room assisting the endoscopist. An endoscopy technician was involved in the care of 13/24 patients (54.2%), a GI fellow in 8/24 (33.3%), an ICU nurse in 3/24 cases (12.5%), and a primary team care member in 1/24 (4.2%). A certified registered anesthetist (CRNA) or anesthesiologist were involved in the care of 12/24 patients (50%) (Fig. 2). Data on possible exposure and infection of endoscopy or anesthesia personnel during these procedures were not collected.

## Discussion

In this consecutive sample of almost 2000 patients hospitalized with COVID-19 across a geographically diverse network of medical centers in North America, only 1.2% of patients underwent endoscopy despite a high prevalence of gastrointestinal symptoms and substantial burden of critical and/or prolonged illness. The majority of endoscopic procedures were performed for either emergency cases (e.g. ongoing GI bleeding, biliary obstruction) or for placement of enteral access tubes. Among those who did undergo endoscopy, the indications and findings were judged more likely to reflect a consequence of overall systemic illness rather than direct viral injury. Endoscopy was performed in a range of locations with variable anesthesia support and staffing. Although the sample was small, there did not appear to be substantively increased procedure or sedation-related risk associated with endoscopic intervention in this patient population.

While 53% of patients in the overall cohort experienced at least one gastrointestinal symptom, most were judged to be mild in nature and they were not associated with more severe outcomes, suggesting that gastrointestinal manifestations are not a principal aspect of this disease in terms of human suffering or resource utilization [2]. The endoscopic findings in this cohort appear to further this observation as only 1 patient was judged by the study personnel to have mucosal pathology that might reasonably be attributed to viral injury rather than systemic illness. Objectively, however, 10 cases did identify an erosive or inflammatory process in the foregut, some fraction of which could conceivably be due to direct viral effect. These upper GI tract findings are generally consistent with those of prior series from Lombardy and New York City, and seem in line with what might be expected in any cohort of severely ill patients. In contrast, however, we did not observe inflammatory pathology in the colon of any patient. While mucosal injury is a hallmark of many viral gastroenteridities, our findings, along with those of prior series, provide growing reassurance that SARS-CoV-2 does not appear to behave as a highly invasive and injurious pathogen to gastrointestinal mucosa.

We found that only 2 of 13 patients who underwent endoscopy for GI bleeding (8.3%; 0.1% of overall cohort) required endoscopic hemostatic therapy (one hemostatic clip placement for duodenal angiodysplasia and one esophageal variceal band ligation). Only six cases—among 878 patients who were admitted to the ICU—were required for enteral access and only 2 ERCPs were necessary for biliary obstruction. The majority of cases (17/27, 63%) were performed for diagnostic purposes and did not require an intervention. This low burden of endoscopic intervention, in the context of the hypothesized increased transmission risk to health care workers involved in the endoscopy, suggests that conservative (non-endoscopic) management of most COVID-19 patients is reasonable. This assertion, however, is tempered by judicious use of endoscopy throughout the pandemic, which may have led to underestimation of GI pathologies that might meaningfully benefit from endoscopic intervention.

In this series, we present data on staffing and location of procedures and anesthesia utilization, which have not been previously reported. Given the resource limitations that have characterized the pandemic, we observed that skeleton teams were included in most procedures; typically, only the endoscopist and the GI nurse, with a GI technician involved in approximately half of cases. A GI fellow was involved in a minority of cases, in line with previously reported studies of decreased endoscopic experience of GI fellows during the COVID-19 pandemic [11]. Furthermore, we observed that significant number of procedures were performed in the ICU or endoscopy unit. Early in the pandemic, most institutions did not mandate endoscopy in a negative pressure room or specialized COVID suite. We also observed that 50% of cases were performed with anesthesiology support. Some studies suggest a higher risk of aspiration events associated with deep sedation (0.22% with anesthesia services undergoing colonoscopy vs 0.16% without anesthesia services undergoing colonoscopy) [12, 13], which may be an important consideration in patients with COVID-19 who often experience respiratory compromise and mental status changes. However, within the limitation of our small sample, we did not observe a substantial difference in adverse events according to form of sedation in our very ill subgroup of patients.

The limitations of this study have been reported previously [2] and include its retrospective design and reliance on medical records review rather than direct patient interviews. Additionally, because it was conducted in North America, the results may be less generalizable to other geographic locations with more limited resources. For this particular secondary analysis, the major limitation is the small sample of patients who underwent endoscopy. This was likely due to the higher threshold adopted for endoscopy during the pandemic and the relatively mild nature of GI manifestations that did not warrant endoscopic evaluation. However, the well-defined and systematic sampling frame in this study is unique and critical to accurately estimating the burden of endoscopic utilization in this patient population. These findings may help inform endoscopic research allocation in the event of ongoing or future regional surges. Furthermore, the size of the overall cohort, the rigorous approach to data collection and verification, and the broad distribution of sites in the study network, all increase the precision of our findings relative to prior studies exploring this topic. Additionally, we were unable to compare our COVID positive patients to COVID negative controls. Lastly, the lack of histologic and microbiologic analysis likely causes us to miss the histologic disease that may very well be caused by virologic injury as our focus was on endoscopic findings.

## Conclusions

In sum, we observed that only 24 of 1992 patients hospitalized with COVID-19 underwent endoscopy and only 10 of these required a clinically meaningful intervention for either bleeding [2], enteral access [6], and biliary obstruction [2]. The majority of endoscopic findings were judged to be related to critical and/or prolonged illness rather than direct viral injury. Variable procedure locations, staffing, and anesthesia utilization were observed.

## Data Availability

The datasets used and/or analyzed during the current study are available from the corresponding author on reasonable request.

## Abbreviations

SARS-CoV-2: Severe acute respiratory syndrome coronavirus 2
GI: Gastrointestinal
US: United States
BMI: Body Mass Index
ERCP: Endoscopic retrograde cholangiopancreatography
OGT: Orogastric tube
ICU: Intensive care unit
CRNA: Certified registered Nurse Anesthetist
